# Naringenin Loaded Hydrogel Supports Wound Repair in a Cell Model of Diabetic Skin

**DOI:** 10.1007/s11095-026-04029-z

**Published:** 2026-01-31

**Authors:** Mandeep Kaur Marwah, Yukta Sameer Hindalekar, Karan Rana, Hala Shokr, Mohamad Anas Al Tahan, Lissette Sanchez-Aranguren, Maymunah Sarr, Rosie Kainth, Parmida Babaei, Humaa Asif, Shakil Ahmad, Anisa Mahomed

**Affiliations:** 1 Aston Medical School, College of Health and Life Sciences, Aston University, Birmingham, UK; 2 Aston Research Centre for Health in Ageing, Aston University, Birmingham, UK; 3 School of Biosciences, College of Health and Life Sciences, Aston University, Birmingham, UK; 4 Pharmacy Division, School of Health Sciences, Faculty of Biology, Medicine and Health, The University of Manchester, Manchester, UK; 5 Chemical Engineering and Biotechnologies, College of Engineering and Physical Sciences, Aston University, Aston Triangle, Birmingham, B4 7ET UK

**Keywords:** Controlled-release, Diabetic wound healing, Hydrogel, Inflammation, Naringenin biological activity, Topical drug delivery

## Abstract

**Introduction:**

Diabetic foot ulcers are a major complication of diabetes, driven by inflammation, oxidative stress, and poor vascular function. Naringenin, a citrus flavonoid, addresses these factors but has low solubility and stability. We developed a Na-AMPS hydrogel dressing to enhance its delivery under diabetic-like conditions.

**Methods:**

A Na-AMPS hydrogel containing 0.02%(w/w) naringenin was formulated and assessed for rheological and adhesive properties, drug release, and biological activity in HUVEC and HDFa cells. Cytotoxicity (XTT), reactive oxygen species (ROS), mitochondrial membrane potential (TMRM), cytokine levels (IL-6, IL-8, MMP-9, TGF-β), and wound closure (scratch assay) were measured.

**Results/Discussion:**

Naringenin modestly reduced the hydrogel elastic modulus (15,791.5 ± 1965 Pa at 30 Hz) without affecting adhesion. Release studies showed rapid drug release from solution but sustained release from hydrogels (17.88 ± 2.61% over 24 h). Under hyperglycaemic and pro-inflammatory conditions, naringenin significantly decreased ROS in HUVECs (41,030.58 ± 2737 to 31,778.74 ± 1822 AU; *p* < 0.001) and HDFa cells (38,188.13 ± 4593 to 29,950.94 ± 1426 AU; *p* < 0.05). Naringenin improved mitochondrial membrane potential in both cell types (*p* < 0.05–0.01) and attenuated pro-inflammatory cytokines. IL-6 decreased in HUVECs (39.40 ± 5.02 to 27.15 ± 3.10 pg/mL; *p* < 0.01) and HDFa cells (40.05 ± 2.23 to 16.41 ± 1.27 pg/mL; *p* < 0.0001). In HDFa’s, MMP-9 was reduced (403.43 ± 18.70 to 195.33 ± 11.02 pg/mL; *p* < 0.0001), while in HUVECs, wound closure was enhanced.

**Conclusion:**

Naringenin-loaded Na-AMPS hydrogels demonstrated sustained release, suitable mechanical properties, and significant antioxidant, anti-inflammatory, and wound healing effects. These findings highlight their therapeutic potential for diabetic wounds treatment.

**Supplementary Information:**

The online version contains supplementary material available at 10.1007/s11095-026-04029-z.

## Introduction

Diabetic foot ulcers represent a significant health issue globally, with substantial economic impact on healthcare systems worldwide. Such wounds commonly arise from chronic diabetes-related complications such as prolonged inflammation [1], impaired vascular function [2], and excessive production of reactive oxygen species (ROS) [3], all of which contribute to poor wound healing, tissue breakdown, and increased susceptibility to infection—often culminating in prolonged hospitalisation and an elevated risk of lower-limb amputation. Treating diabetic foot ulcers incurs considerable financial burdens, with expenditures in the United States nearing $13 billion and approximately £456 million in the UK [4, 5]. The annual incidence of diabetic foot ulcers in diabetic patients is ca. 2.5% to 5%, while the lifetime risk is estimated at 15% to 20% [6, 7]. Despite their prevalence and severity, current treatment options for diabetic foot ulcers remain limited, often addressing symptoms rather than underlying pathophysiological mechanisms [8]. Therefore, there is an urgent need for the development of targeted, multifunctional interventions that can modulate inflammation, oxidative stress, and impaired tissue remodelling to promote effective wound healing in diabetic patients.

One potential solution is naringenin, a naturally occurring flavonoid found in citrus fruits, known for its potent anti-inflammatory, antioxidant, and tissue-protective effects demonstrated in various preclinical studies [9, 10]. Notably, recent in vivo evidence supports naringenin’s direct efficacy in wound healing. In a diabetic mouse model induced by streptozotocin, topical naringenin significantly accelerated wound closure, reduced inflammatory cytokines, promoted macrophage M2‑polarization and efferocytosis, and enhanced collagen deposition compared to untreated diabetic wounds [11]. Moreover, network‑pharmacology and in vivo wound studies demonstrated that naringenin inhibits wound inflammation and oxidative stress in chronic wound models, supporting its wound‑healing potential beyond antioxidant activity [12]. However, despite its therapeutic promise, naringenin’s clinical application is limited by poor water solubility and chemical instability which pose significant challenges for effective delivery and sustained activity at wound sites [13]. Addressing these limitations through innovative drug delivery systems is critical to harnessing naringenin’s full potential as a treatment for diabetic foot ulcers. Loading naringenin into topical hydrogels represents a promising strategy to overcome these barriers, as similar flavonoids, including quercetin and curcumin have shown improved solubility, stability, and local bioavailability when delivered via hydrogel or nanogel systems [14, 15].

Hydrogels are crosslinked polymer networks formed from hydrophilic homopolymers or copolymers, capable of absorbing large amounts of water due to their highly swollen structures and unique physicochemical properties [16]. Hydrogels synthesised via radical polymerisation of 2-acrylamido-2-methylpropane sulfonic acid (AMPS) or its sodium salt (Na-AMPS) in aqueous solution have gained significant attention for a broad range of medical applications, including dermal dressings [17, 18]. Na-AMPS is a highly hydrophilic monomer containing a sulfonate group which offers Na-AMPS-based hydrogels several advantages for the transdermal delivery of hydrophobic drugs. In the first instance its hydrophilicity enhances skin hydration thus softening the stratum corneum and improving drug permeation. The negatively charged sulfonate group enables electrostatic interactions that can stabilise drug carriers like micelles or nanoparticles within the hydrogel [19, 20]. Such hydrogels have demonstrated potential for biocompatibility, are chemically stable and possess favourable mechanical and rheological properties for dermal applications. Importantly, their network structure is tuneable, which enables drug release kinetics to be precisely controlled while maintaining structural integrity and patient comfort [18, 21]. These hydrogels exhibit excellent swelling properties, mopping up excess wound exudate whilst providing a moist environment conducive to wound healing [18, 22]. The ability of a Na-AMPS formulated hydrogel to host hybrid drug delivery systems is thus a promising strategy to topically deliver naringenin to diabetic foot ulcers. By incorporating hydrophobic drug carriers into an Na-AMPS hydrogel matrix, the naringenin solubility barrier can be overcome whilst leveraging skin hydration to improve local drug bioavailability and therapeutic efficacy, thereby expanding the boundaries of current transdermal delivery technologies.

In this study, we developed and characterised a Na-AMPS-based topical gel formulation loaded with naringenin to address the challenges of its poor solubility and stability, while ensuring a sustained release of the active compound. We assessed naringenin release profile from the gel alongside its ability to modulate key pathological features of diabetic wounds—including inflammation, oxidative stress, and tissue remodelling—using in vitro models of human endothelial and dermal fibroblast cells exposed to hyperglycaemic and pro-inflammatory conditions. The novelty of this work lies in combining controlled naringenin delivery with direct functional evaluation of its effects on multiple cellular mechanisms relevant to diabetic wound healing. Our findings aim to provide proof-of-concept for this multifunctional delivery system as a promising therapeutic strategy to enhance local drug bioavailability and promote healing in diabetic foot ulcers.

## Methods

### Materials

1-Hydroxycyclohexyl phenyl ketone (Omnirad 184, catalogue # 405,612), sodium 2-acrylamido-2-methylpropane sulfonate (Na-AMPS, catalogue # 655,821), Naringenin (catalogue # N5893), and propylene glycol (catalogue #W294004) were obtained from Sigma-Aldrich (Dorset, England). Polyethylene glycol 400 (PEG-400) diacrylate (SR344) was obtained from Arkema. Poloxomer 184 (Pluracare® L64G) was obtained from BASF. All other reagents including isopropanol, ethanol and phosphate-buffered saline were obtained from Fisher Scientific. Ultrapure water was obtained from a Milli-Q purification system (Millipore, Billerica, MA, US). Tumour necrosis factor-alpha (TNF-α) (Cat. No. 210-TA-005) was purchased from R&D Systems, Abingdon, UK.

### Gel Formulation

The adhesive hydrogel formulation was primarily composed of sodium 2-acrylamido-2-methylpropane sulfonate (Na-AMPS), which functioned as the principal monomer and provided intrinsic hydrophilicity, accounting for 75.68% (w/w) of the total composition. Propylene glycol was incorporated at a final concentration of 5.1% (w/w) to enhance moisture retention and plasticity. To facilitate the solubilisation of the hydrophobic active compound, naringenin, a non-ionic surfactant, Poloxamer 184, was included at 18.1% (w/w). Naringenin was subsequently added at a final loading of 0.02% (w/w). Photopolymerisation was initiated using a proprietary blend of Omnirad 184 as the photoinitiator and (PEG-400) diacrylate as the cross-linking agent, with a combined loading of 1.1% (w/w). The formulation was protected from ambient light by wrapping the container in aluminium foil and mixed thoroughly to ensure homogeneity. The solution was then degassed under a nitrogen stream for 2 min to eliminate entrapped air. Following degassing, the formulation was cast onto a silicone substrate and cured under ultraviolet (UV) light to form a lightly crosslinked polymeric adhesive hydrogel. Additional water was not added, as excessive water content could compromise the adhesive and mechanical properties of the hydrogel.

### Gel Characterisation

#### Dynamic Shear Rheological Properties

The dynamic shear rheological properties of the hydrogel were examined using a dynamic shear rheometer (Malvern, KINEXUS) in oscillatory mode. The storage modulus (G′), loss modulus (G″) and complex viscosity (η*) were evaluated. The test was conducted within the linear viscoelastic region (LVER), under a strain of 0.02 (2%). Oscillatory frequency sweep tests, from 5 to 30 Hz, were executed utilising a 20 mm diameter plate/plate configuration at 36 ℃, the gap varied according to the thickness of the sample but it was ensured that the normal force was comparable. The tests were performed in triplicate and results are presented as the mean and standard deviation of each measurement.

#### Adhesion Properties of Hydrogel

The adhesive performance of the hydrogels was assessed using an Instron machine with a ball-tack test setup, equipped with a 50 N load cell. Hydrogel samples were cut into 2.5 cm diameter discs and tested for the force required to detach from a solid surface, simulating real-world application and removal. During the test, a 1-inch stainless steel probe was brought into contact with the hydrogel (loading phase), then withdrawn until detachment (unloading phase). The test was conducted with a displacement rate ranging from −0.50 mm/s (compression) to 0.50 mm/s (tension), and a trigger force of 2.5 g was applied to initiate the measurement. The maximum release force required for detachment was recorded, providing a quantitative measure of the adhesive strength. This metric is important for evaluating whether the hydrogel can maintain secure adhesion to biological tissue while still allowing for non-traumatic removal, which is critical for wound care applications.

#### Swelling Behaviour of Gel Formulations

The swelling behaviour of the hydrogels was assessed by completely immersing 1 g hydrogel sample in deionised water at room temperature [23]. At selected time intervals (0–40 min), the samples were removed from the water, gently blotted with filter paper to remove excess surface water, and immediately weighed to determine the swollen weight. The swelling ratio (%) was calculated using the following equation:

$$\% Swelling=\frac{{W}_{f}-{W}_{i}}{{W}_{i}} \times 100$$

where W_i_ is the initial weight of the dry hydrogel, and W_f_ is the weight of the hydrogel at the given time point.

#### *In Vitro* Diffusion of Naringenin from Gel Formulations

To evaluate the release profile of naringenin gel formulations, an in vitro permeable insert model was employed, as previously described [24]. This setup enabled a comparative assessment of naringenin release from the naringenin solution and naringenin gel. A 4 cm^2^ cylindrical cell culture insert (Thincert™, 400 µm pore size) was filled with 1 mL of each test formulation and placed into individual wells of a 6-well Thincert™ plate containing 4 mL of phosphate-buffered saline (PBS) as the receptor medium. Plates were maintained at 35 °C on a shaking platform to simulate physiological diffusion conditions. At predetermined intervals over 6 h, 0.5 mL aliquots were collected and immediately replaced with fresh PBS to maintain sink conditions. Naringenin release was quantified using UV–visible spectroscopy.

### Cell Culture and Treatment

Human Umbilical Vein Endothelial Cells (HUVECs) (PromoCell, Cat. #C-12203) were cultured in complete Endothelial Growth Medium-2 (EGM-2; PromoCell, Cat. #C-22211), supplemented with a growth factor mix comprising fetal calf serum (0.02 mg/mL), epidermal growth factor (5 ng/mL), basic fibroblast growth factor (10 ng/mL), insulin-like growth factor (20 ng/mL), vascular endothelial growth factor (0.5 ng/mL), ascorbic acid (1 μg/mL), heparin (22.5 μg/mL), and hydrocortisone (0.2 μg/mL) (Supplement Kit, PromoCell, Cat. #C-39211) as described previously [25]. Penicillin/streptomycin (5 mL of 1X; Lonza, Cat. #LZDE17-602E) was also added. Cells were maintained at 37 °C in a humidified incubator with 5% CO₂, and the medium was replaced every 48 h. Subculturing was carried out at 70–80% confluency, and cells ≤ 5 passages were used for all experiments.

Human Dermal Fibroblasts (HDFa) (Thermo Fisher, Cat. #C0135C), derived from adult skin, were cultured in Human Fibroblast Expansion Basal Medium (Thermo Fisher, Cat. #M106500) supplemented with Low Serum Growth Supplement (LSGS) (Thermo Fisher, Cat. #S00310). The LSGS supplement included 2% (v/v) fetal bovine serum, hydrocortisone (1 μg/mL), human epidermal growth factor (10 ng/mL), basic fibroblast growth factor (3 ng/mL), and heparin (10 μg/mL).

#### Cytotoxicity Assessment of Naringenin in HUVEC and HDFa Cells

The cytotoxicity of naringenin released from hydrogel formulations on Human Umbilical Vein Endothelial Cells (HUVECs) and Human Dermal Fibroblasts (HDFa) was evaluated using the XTT [2,3-bis-(2-methoxy-4-nitro-5-sulphophenyl)−2H-tetrazolium-5-carboxanilide] assay supplied with 1 mM menadione (Biotium, Cat. #30,007). Cells were seeded at a density of 5 × 10^4^ cells per well in 96-well plates and cultured for 48 h. The permeate obtained from in vitro release studies was quantified using HPLC–UV, and this known concentration was used to prepare the test concentration range. The culture medium was then replaced with 100 µL of fresh medium containing permeate collected from in vitro release studies of naringenin-loaded hydrogels at concentrations ranging from 0.1 to 5 µM. Following a 24-h incubation at 37 °C, 25 µL of XTT/menadione reagent (12.5:1 ratio) was added to each well, and plates incubated for an additional 3 h. Absorbance was measured at 450 nm using a microplate reader (TECAN, Switzerland), and cell viability was determined by analysing absorbance values relative to naringenin concentration compared to control cells with no treatment.

#### Measurement of Intracellular Reactive Oxygen Species (ROS) in HUVEC and HDFa Cells

Intracellular ROS levels were quantified using the DCFDA/H2DCFDA Cellular ROS Assay Kit (Abcam, Cat. #ab113851), following the manufacturer’s protocol with minor modifications. HUVECs and HDFa cells were seeded at a density of 1 × 10^4^ cells per well in black-walled, clear-bottom 96-well plates and allowed to adhere overnight. Cells were then subjected to TNF-α stimulation (5 ng/mL), followed by treatment with or without naringenin gel formulation permeate (0.5 µM) for 24 h [26]. After treatment, cells were washed with 1 × PBS and incubated with 100 µL of 25 µM DCFDA solution in serum-free medium for 45 min at 37 °C in the dark. Fluorescence was measured at excitation/emission wavelengths of 485/535 nm using a Spark® multimode microplate reader (TECAN, Switzerland). Background fluorescence was determined from reference wells containing cells with media only and subtracted from all experimental readings to reduce noise.

#### Assessment of Mitochondrial Membrane Potential Using TMRM Staining

Mitochondrial membrane potential was evaluated using tetramethylrhodamine methyl ester (TMRM) staining. A 100 nM TMRM solution was prepared in serum-free medium appropriate for each cell type. Following treatment with or without naringenin gel formulation permeate (0.5 µM) for 24 h, culture medium was aspirated, and cells washed with PBS. Cells were then incubated with 50 µL of TMRM solution for 30 min at 37 °C in the dark. After incubation, the staining medium was aspirated, and cells bathed in HEPES Buffer. Fluorescence imaging was performed immediately using a fluorescence microscope. Fluorescence was then measured with a Spark® multimode microplate reader at excitation/emission wavelengths of 548/573 nm (TECAN, Switzerland).

#### Assessment of Anti-Inflammatory and Matrix Remodelling Effects of Naringenin Permeate in HUVEC and HDFa Cells

To assess the anti-inflammatory and matrix remodelling regulatory potential of naringenin permeate, secretion levels of interleukin-6 (IL-6), interleukin-8 (IL-8), matrix metalloproteinase-9 (MMP-9), and transforming growth factor-beta (TGF-β) were quantified in cell culture supernatants using Human DuoSet enzyme-linked immunosorbent assay (ELISA) kits (R&D Systems; Cat. #DY202, #DY208, #DY911, and #DY240, respectively). HUVEC and HDFa cells were seeded into 12-well plates and allowed to adhere overnight under standard culture conditions. Inflammatory stimulation was induced using TNF-α; 5 ng/mL for 3 h, followed by medium replacement and an additional 24-h incubation to establish an inflammatory baseline. Cells were then washed with PBS and treated with naringenin hydrogel permeate (0.5 µM) for 24 h. Conditioned media was subsequently collected and stored at − 80 °C until cytokine and matrix regulator quantification via ELISA, performed in accordance with the manufacturer’s protocols.

#### Evaluation of Wound Healing Potential of Naringenin by Scratch Assay

The wound healing potential of naringenin was assessed using a scratch assay in HUVECs. HUVECs were chosen to specifically evaluate endothelial cell migration, a key component of angiogenesis during wound healing. Cells were seeded in 24-well plates at a density of 5.0 × 10^4^ cells per well and cultured to confluence. Except for control wells, cells were exposed to TNF-α; 5 ng/mL for 3 h. Following a linear scratch was created in the monolayer using a sterile 200 μL pipette tip (time 0 h), after which detached cells and debris were removed by washing twice with warm PBS. Naringenin permeate (0.5 µM) was then applied to the cells for 24 h. Images of wound closure were captured at 24 h using a Nikon Eclipse Ti-E phase-contrast inverted microscope (Nikon Instruments Inc., Melville, NY, USA). The percentage of wound closure was quantified using the ImageJ Wound Healing Tool plugin (Montpellier Resources Imagerie, Montpellier, France) and expressed relative to the initial wound area at 0 h.

### Statistical Analysis

Results are expressed as mean ± standard deviation. Statistical analysis was performed using GraphPad Prism version 9.4.1 (La Jolla, CA, USA). Data were evaluated using unpaired t-tests, as well as one-way and two-way ANOVA followed by post-hoc analyses, with *p* < 0.05 considered statistically significant.

## Results

### Dynamic Shear Rheological Properties

**Fig. 1:**
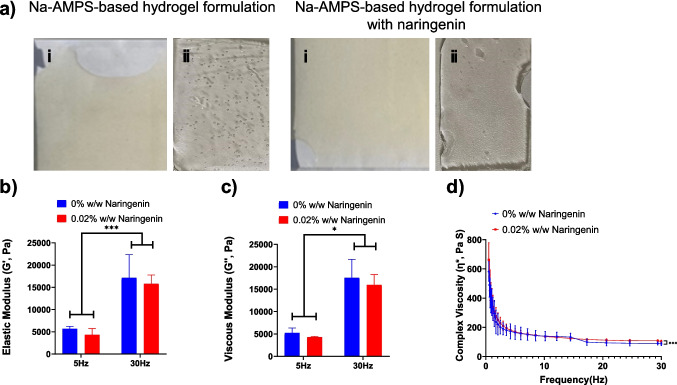
(**a**) Representative images of the Na-AMPS-based hydrogel formulation without Naringenin and with 0.02% (w/w) naringenin, both with the (i) backing layer and (ii) with backing layer removed showing visual homogeneity, transparency and uniformity. (**b**) Elastic modulus (G', Pa) and (**c**) viscous modulus (G'', Pa) of the gels measured at 5 Hz and 30 Hz, demonstrating frequency-dependent increases in both parameters. (**d**) Complex viscosity (η*) profiles of adhesive hydrogels loaded with naringenin, revealing characteristic shear-thinning behaviour. Hydrogels were composed of Na-AMPS (75.68% w/w), propylene glycol (5.1% w/w), Poloxamer 184 (18.1% w/w) and naringenin (0.02% w/w). Photopolymerisation was carried out using Omnirad 184 and PEG-400 diacrylate (1.1% w/w total). Data represent mean ± SD, *n* = 4. Statistical significance: *p < 0.05, ***p < 0.001.

Hydrogels with and without 0.02% (w/w) naringenin were successfully formulated, appearing uniform and visually clear (Fig. 1a). Rheological analysis was used to assess mechanical stability of the hydrogel network, focusing on the elastic modulus (G′), viscous modulus (G″), and complex viscosity (η*). The elastic modulus reflects the material’s solid-like behaviour, while the viscous modulus indicates its liquid-like response. Complex viscosity represents the overall resistance to deformation under oscillatory shear. The elastic modulus (G') of the gels was assessed at 5 Hz and 30 Hz to evaluate the impact of Naringenin incorporation on gel stiffness (Fig. 1b). For gels without naringenin (0% w/w), the elastic modulus was 5672 ± 565 Pa at 5 Hz, increasing to 17,153 ± 5175 Pa at 30 Hz, reflecting the expected frequency-dependent increase in stiffness. In contrast, gels loaded with 0.02% w/w naringenin exhibited a slightly lower modulus, measured at 4326 ± 1403 Pa at 5 Hz and 15,791.5 ± 1965 Pa at 30 Hz. These findings indicate while gel stiffness increases with frequency, the addition of naringenin modestly reduces the elastic modulus, suggesting a slight softening effect on the gel network. The viscous modulus (G'') of the gels was also evaluated at 5 Hz and 30 Hz (Fig. 1c). At 5 Hz, gels without naringenin (0% w/w) exhibited a viscous modulus of 5198.67 ± 1113.8 Pa, while gels with 0.02% w/w naringenin showed a slightly lower value of 4287 ± 188 Pa. At 30 Hz, the viscous modulus increased as expected with frequency, reaching 17,543.33 ± 4111.9 Pa for the unloaded gels and 15,948.5 ± 2315 Pa for the naringenin-loaded gels. These results demonstrate while frequency elevates viscous behaviour, inclusion of naringenin slightly reduces gel viscosity, consistent with the observed reduction in elastic modulus.

The complex viscosity (η*) of the hydrogels across increasing frequencies demonstrated a characteristic shear-thinning profile (Fig. 1d). As frequency increased from 0.5 Hz to 30 Hz, η* decreased significantly for both unloaded and naringenin-loaded hydrogels, indicating reduced viscosity under higher shear stress. This behaviour is desirable for adhesive hydrogel applications, as it facilitates material spreading during application while allowing the gel to retain position once stress is removed. At 0.5 Hz, the unloaded gel (0% w/w naringenin) exhibited a complex viscosity of 581.9 ± 67 Pa·s, decreasing to 87.2 ± 12.3 Pa·s at 30 Hz. Similarly, the 0.02% w/w naringenin gel showed a higher initial η* of 662.4 ± 117 Pa·s at 5 Hz, reducing to 104 ± 7.7 Pa·s at 30 Hz. These reductions were statistically significant (*p* < 0.001), confirming that naringenin inclusion does alter the overall shear-thinning behaviour of the hydrogel, while slightly increasing the initial viscosity at lower frequencies.

### Adhesion Properties

The hydrogel adhesive performance was assessed using a ball-tack test, which measures the force required to detach the hydrogel from a solid surface, simulating real-world application and removal. The test involves loading (bringing a 1 inch steel ball probe into contact with the hydrogel) followed by unloading (withdrawing the probe until detachment). Three representative force–displacement curve for the naringenin loaded gel is shown in Supplementary Fig. 1 (Appendix A). During loading, compressive force is applied until a trigger threshold is reached. The maximum pull-off force recorded during unloading reflects the adhesive strength by capturing the peak tensile force required to overcome adhesive and cohesive forces. No significant difference was observed between 0% w/w and 0.02% w/w naringenin formulations, (2.62 ± 0.14 and 3.04 ± 0.32 gf respectively) indicating that within this concentration range, the active does not affect hydrogel adhesion properties (Table I).

**Table I:** Adhesion properties of Na-AMPS hydrogel formulations measured using a 1-inch stainless steel ball in a ball-tack test. Maximum and minimum pull-off forces were recorded during unloading and loading, respectively. Naringenin concentrations indicate % w/w incorporated into the hydrogel

Formulation (% w/w Naringenin in hydrogel)	Maximum force (gf)	Minimum force (gf)
0% w/w Naringenin	2.62 ± 0.14	−3.17 ± 0.08
0.02% w/w Naringenin	3.04 ± 0.32	−2.83 ± 0.2

The minimum force recorded during the loading phase reflects the initial contact resistance as the probe compresses the hydrogel surface. This indicates how readily the adhesive material conforms to the contacting surface. The minimum forces during the loading phase were − 3.17 ± 0.08 gf for 0% naringenin and − 2.83 ± 0.2 gf for 0.02% naringenin gels (Table I). These values show no significant difference, indicating that naringenin concentration does not affect the initial contact resistance or how the hydrogel conforms to the surface.

### Swelling Behaviour of Hydrogel Formulations

**Fig. 2:**
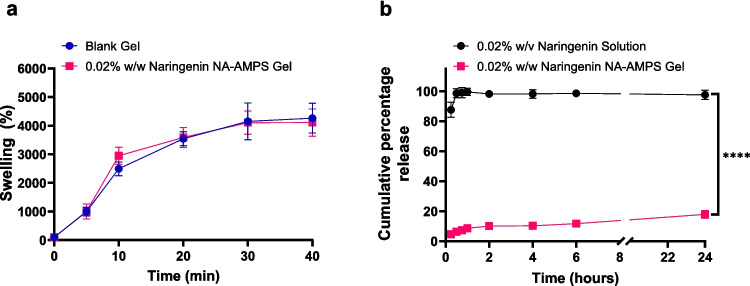
(**a**) Swelling behaviour of blank and naringenin-loaded Na-AMPS hydrogels over 40 min in deionised water. Both formulations reached equilibrium swelling within 30 min, with no significant difference between them. (**b**) In vitro cumulative release profiles of naringenin from solution and Na-AMPS-based gel formulations over 24 h. Hydrogels were composed of Na-AMPS (75.68% w/w), propylene glycol (5.1% w/w), Poloxamer 184 (18.1% w/w) and naringenin (0.02% w/w). Photopolymerisation was carried out using Omnirad 184 and PEG-400 diacrylate (1.1% w/w total). A dialysis system was used to assess in vitro drug release. Data represent mean ± SD, *n* = 4. Statistical significance: ****p < 0.0001.

The swelling capacity of blank and naringenin-loaded Na-AMPS hydrogels was evaluated over 40 min in deionised water at room temperature (Fig. 2a). Both formulations exhibited rapid water uptake within the first 10 min, followed by a slower increase approaching equilibrium. Maximum swelling was observed at 30 min for both blank (4151.67 ± 645.08%) and naringenin-loaded gels (4105.67 ± 406.02%), with no statistically significant difference between the two formulations (*p* > 0.05). After 30 min, swelling plateaued, with only minor changes at 40 min, indicating that equilibrium swelling had been reached. These results suggest that incorporation of 0.02% w/w naringenin did not significantly alter the hydrogel’s water-absorbing capacity.

### *In Vitro* Release of Naringenin from Hydrogel Formulations

To evaluate the release characteristics of naringenin, comparative in vitro studies were conducted using both solution and gel formulations over a 24-h period (Fig. 2b). Naringenin release from solution achieved 98.56 ± 3.01% within 30 min, indicating rapid and complete release. In contrast, the naringenin-loaded gel exhibited a sustained release profile, reaching only 17.88 ± 2.61% over 24 h. The release profiles over 24 h were significantly different (*p* < 0.0001), demonstrating the gel’s capacity for prolonged and controlled release compared to the rapid release from solution.

### Cytotoxicity Assessment of Naringenin in HUVEC and HDFa Cells

**Fig. 3:**
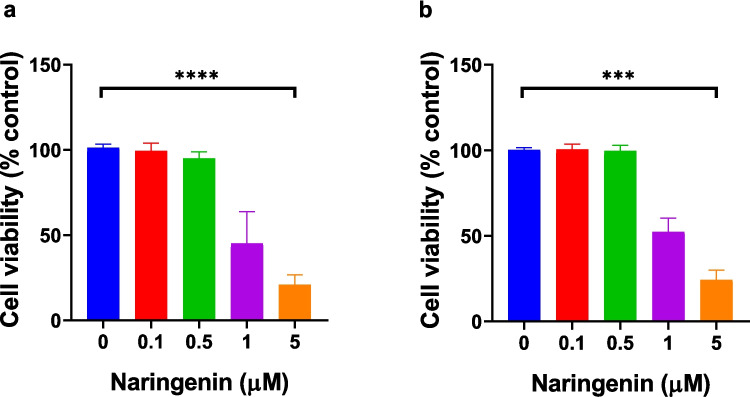
Cytotoxicity of naringenin in HUVEC and HDFa cells assessed by XTT assay following 24-h exposure. Cells were treated with discrete concentrations of naringenin (0, 0.1, 0.5, 1, and 5 μM) as the permeate collected from in vitro hydrogel release studies, and cell viability (%) was measured. The blue line at 0 µM represents untreated control cells. Naringenin showed no significant cytotoxicity at concentrations up to 0.5 μM in either (**a**) HUVEC or (**b**) HDFa cells. However, a significant dose-dependent decrease in viability was observed at 1 μM and 5 μM (*p < 0.0001), indicating cytotoxic effects at higher concentrations. Data represent mean ± SD, *n* = 6. Statistical significance: ***p < 0.001, ****p < 0.0001.

Cytotoxicity of permeate solution collected from in vitro naringenin release studies was evaluated in HUVEC and HDFa cells using the XTT assay after 24-h exposure to concentrations between 0.1 and 5 μM (Fig. 3). In HUVECs, cell viability remained above 90% at 0.1 μM (99.45 ± 4.47%) and 0.5 μM (94.89 ± 3.86%), with no significant cytotoxicity observed at these concentrations. However, a substantial, concentration-dependent reduction in viability was observed at 1 μM (45.10 ± 18.76%) and 5 μM (20.89 ± 5.87%), with differences statistically significant (*p* < 0.0001). Similarly, HDFa cells exhibited high viability at lower concentrations (100.47 ± 3.13% at 0.1 μM and 99.56 ± 3.31% at 0.5 μM), comparable to the untreated control (100.31 ± 1.29%). Marked cytotoxic effects were observed at higher doses, with viability declining to 52.29 ± 8.03% at 1 μM and 24.29 ± 5.71% at 5 μM (*p* < 0.0001). These results indicate that naringenin is non-cytotoxic at submicromolar concentrations in both endothelial and dermal fibroblast cells, but higher concentrations (≥ 1 μM) significantly reduce cell viability in a dose-dependent manner.

### Modulation of Intracellular Reactive Oxygen Species (ROS) by Naringenin in HUVEC and HDFa Cells

**Fig. 4:**
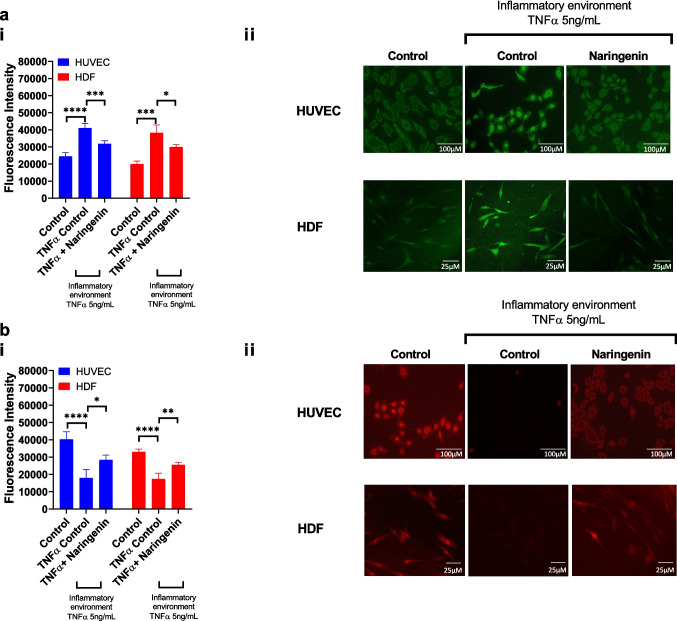
Naringenin-loaded hydrogel permeate reduce oxidative stress and preserve mitochondrial membrane potential in TNF-α-stimulated HUVEC and HDFa cells. (**a**) Intracellular ROS levels were quantified using fluorescence intensity (ai), showing significant ROS elevation in TNF-α-stimulated cells compared to untreated controls. Treatment with naringenin-loaded hydrogels significantly attenuated ROS levels in both HUVEC and HDFa cells. (a ii) Representative fluorescence images illustrating ROS levels across groups. (**b**) Mitochondrial membrane potential was assessed using TMRM staining. (b i) Quantitative fluorescence intensity data demonstrated mitochondrial depolarisation in TNF-α-stimulated cells, which was partially restored by naringenin treatment. (b ii) Representative TMRM fluorescence images. Data represent mean ± SD (*n* = 6). Statistical significance: * *p* < 0.05, ** *p* < 0.01, ***p < 0.001, ****p < 0.0001.

Intracellular ROS levels were quantified to assess oxidative stress in both HUVEC and HDFa cells under hyperglycaemic and pro-inflammatory conditions, and the antioxidant effect of naringenin (Fig. 4a). In HUVECs, baseline ROS levels were 24,412.08 ± 2,213 AU under control conditions. Exposure to a hyperglycaemic and pro-inflammatory environment significantly elevated ROS to 41,030.58 ± 2,737 AU (*p* = 0.0001 vs. control), indicating substantial oxidative stress. Naringenin treatment under these conditions significantly reduced ROS production to 31,778.74 ± 1,822 AU (*p* = 0.001 vs. untreated inflammatory condition), demonstrating a moderate antioxidant effect. In HDFa cells, ROS levels were 19,911.02 ± 1,828 AU under control conditions, increasing significantly to 38,188.13 ± 4,593 AU in the hyperglycaemic and pro-inflammatory group (*p* = 0.001 vs. control). Naringenin treatment significantly attenuated ROS production, reducing it to 29,950.94 ± 1,426 AU (*p* = 0.05 vs. untreated inflammatory condition), indicating a statistically significant antioxidant response.

### Mitochondrial Membrane Potential Preservation by Naringenin

The effect of naringenin permeate on mitochondrial membrane potential was assessed by quantifying TMRM fluorescence in HUVEC and HDFa cells under TNF-α-induced inflammatory conditions (Fig. 4b). In HUVECs, TNF-α significantly reduced TMRM fluorescence intensity compared to untreated controls (17,865 ± 4,952 vs. 40,243 ± 4,367 A.U.; *p* < 0.0001), indicating marked mitochondrial depolarisation. Treatment with Naringenin (10 µM) partially restored TMRM fluorescence to 28,367 ± 2,811 A.U. (*p < 0.05 vs. TNF-α), suggesting a protective effect on mitochondrial function.

In HDFas, a similar trend was observed. TNF-α reduced fluorescence from 33,048 ± 1,589 A.U. in controls to 17,308 ± 3,408 A.U. (*p* < 0.0001). Naringenin co-treatment significantly improved mitochondrial membrane potential, increasing fluorescence to 25,448 ± 1,484 A.U. (*p* < 0.01 vs. TNF-α). These results support the role of Naringenin in mitigating TNF-α-induced mitochondrial dysfunction in both endothelial and dermal fibroblast cells.

### Assessment of Anti-Inflammatory and Matrix Remodelling Effects of Naringenin Hydrogel in HUVEC and HDFa Cells

**Fig. 5:**
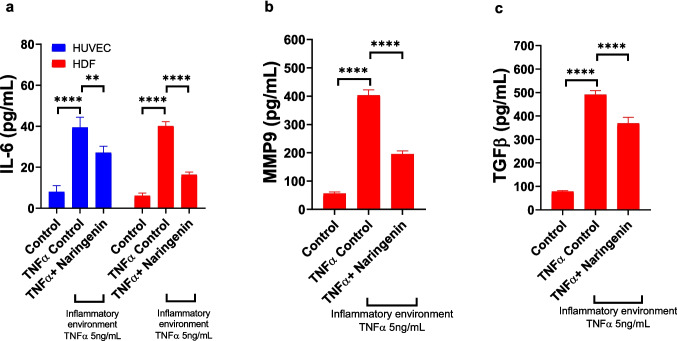
Anti-inflammatory effects of naringenin gel permeate in HUVEC and HDFa cells under inflammatory conditions. (**a**) IL-6 secretion was significantly increased in both cell types under inflammatory conditions (TNF-α 5 ng/mL), and naringenin gel treatment significantly reduced IL-6 levels. (**b**) In HDFa cells, MMP-9 levels were elevated under inflammatory conditions and were significantly reduced following treatment with naringenin gel. (**c**) TGF-β secretion was also increased under inflammatory conditions and moderately suppressed by naringenin gel treatment. Data are presented as mean ± SD, *n* = 4. Statistical significance: *p < 0.05, **p < 0.01, ***p < 0.001, ****p < 0.0001.

IL-6 concentrations were measured via ELISA to assess the inflammatory response in both HUVEC and HDFa cells under hyperglycaemic and pro-inflammatory conditions, as well as the modulatory effect of naringenin (Fig. 5a). In HUVECs, baseline IL-6 levels under control conditions were 8.00 ± 3.07 pg/mL. Upon exposure to a hyperglycaemic and pro-inflammatory environment, IL-6 secretion increased significantly to 39.40 ± 5.02 pg/mL (*p* = 0.0001 vs. control), indicating a marked pro-inflammatory response. Treatment with naringenin under the same inflammatory conditions significantly reduced IL-6 levels to 27.15 ± 3.10 pg/mL (*p* = 0.01 vs. untreated inflammatory condition). In HDFa cells, control conditions yielded IL-6 levels of 6.16 ± 1.21 pg/mL, which rose substantially to 40.05 ± 2.23 pg/mL under hyperglycaemic and pro-inflammatory conditions (*p* = 0.0001 vs. control). Naringenin treatment significantly reduced IL-6 secretion to 16.41 ± 1.27 pg/mL (*p* = 0.0001 vs. untreated inflammatory conditions). These findings indicate naringenin effectively attenuates IL-6 production in both endothelial and dermal fibroblast cells subjected to diabetic-like inflammatory stress.

MMP-9 levels were quantified by ELISA to evaluate extracellular matrix remodelling in HDFa cells exposed to hyperglycaemic and pro-inflammatory conditions, and the modulatory effect of naringenin (Fig. 5b). Under control conditions, MMP-9 expression was low, with an average of 56.33 ± 5.42 pg/mL. Stimulation with a hyperglycaemic and pro-inflammatory environment significantly elevated MMP-9 secretion to 403.43 ± 18.70 pg/mL (*p* < 0.0001), indicating pronounced matrix-degrading activity. Treatment with naringenin under these conditions significantly reduced MMP-9 levels to 195.33 ± 11.02 pg/mL (*p* < 0.0001), demonstrating a strong anti-remodelling and anti-inflammatory effect. TGF-β levels were measured by ELISA to assess the fibrotic and pro-healing signalling response of HDFa cells under diabetic-like inflammatory conditions and following treatment with naringenin (Fig. 5c). Under control conditions, TGF-β expression was relatively low, at 77.75 ± 4.35 pg/mL. Exposure to a hyperglycaemic and pro-inflammatory environment resulted in a substantial increase in TGF-β secretion to 491.25 ± 17.04 pg/mL (*p* = 0.0001), consistent with excessive pro-fibrotic signalling. Treatment with naringenin significantly reduced TGF-β levels to 368.75 ± 25.53 pg/mL (*p* < 0.0001), indicating a partial but significant attenuation of the fibrotic response.

### Evaluation of Wound Healing Potential of Naringenin by Scratch Assay

**Fig. 6:**
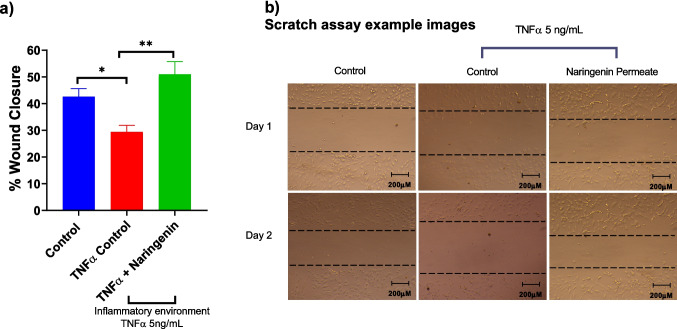
Naringenin permeate restores TNF-α–impaired cell migration in the scratch wound healing assay. HUVECs were exposed to TNF-α (5 ng/mL) for 24 h to inhibit cell migration, followed by treatment with naringenin. (**a**) Quantitative analysis of percentage wound closure. (**b**) Representative images of scratch wounds at 0 and 24 h for control, TNF-α, and naringenin + TNF-α groups. Data represent mean ± SEM, *n* = 3. Statistical significance is indicated as follows: * *p* < 0.05, ** *p* < 0.01, ***p < 0.001; ****p < 0.0001.

HUVECs were used to assess endothelial cell migration, a key step in angiogenesis during wound healing. In the scratch wound healing assay (Fig. 6), TNF-α significantly reduced wound closure compared with the untreated control, decreasing migration from 42.55 ± 3.04% to 29.39 ± 2.48% (*p* < 0.01). Treatment with naringenin markedly improved wound closure to 50.96 ± 4.75%, which was significantly higher than both the TNF-α group (*p* < 0.001) and the untreated control (*p < 0.05). These findings suggest naringenin effectively counteracts TNF-α–induced inhibition of endothelial cell migration and promotes enhanced wound repair.

## Discussion

The development of effective hydrogel-based wound dressings requires a careful balance of mechanical stability, bioadhesion, drug release behaviour, and biological activity to support tissue repair. In this study, we evaluated a Na-AMPS-based hydrogel formulation incorporating naringenin, an antioxidant flavonoid, for its suitability as an adhesive, bioactive wound dressing. A comprehensive characterisation was undertaken, including rheological and adhesive performance, in vitro drug release, cytotoxicity, and wound healing assay. Taken together, the results highlight the potential of this gel to provide sustained naringenin delivery while maintaining favourable mechanical and biological properties suitable for topical application.

The assessment of elastic (G') and viscous (G'') moduli provides critical insight into the mechanical behaviour of hydrogels, particularly for adhesive wound dressings where a balance of rigidity and flexibility is essential. A higher elastic modulus compared to the viscous modulus indicates a predominantly solid-like behaviour, which is necessary for maintaining the dressing’s shape, structural integrity, and consistent adhesion under mechanical stress [27–29]. Conversely, some viscous behaviour is beneficial to ensure conformability to the wound area and patient comfort. In this study, both naringenin-loaded and unloaded gels exhibited G' values greater than G'' across tested frequencies, confirming the gels maintain a solid-like, elastic character suitable for adhesive applications. This is comparable to previous studies where PVA and PVA-gellan gum composite hydrogels [28] as well as poly(AAm-co-SVBA) hydrogels [30] also exhibited G' values greater than G'', which is indicative of a stable structure and elasticity. Although our gels did not incorporate gellan gum, the presence of naringenin did not compromise the solid-like character of the gels. However, while the relatively close values of G′ and G″ in our formulation suggest a viscoelastic balance beneficial for spreadability and comfort, this may limit mechanical robustness under dynamic conditions. Further optimisation is warranted to increase G′ relative to G″. Future work may explore modifying crosslink density, incorporating reinforcing biopolymers, or adjusting the polymer blend to enhance elasticity without compromising biocompatibility or drug release characteristics.

As expected, increasing frequency from 5 to 30 Hz elevated both G’ and G’’, reflecting increased hydrogel stiffness and resistance to deformation under dynamic conditions. Incorporation of 0.02% w/w naringenin resulted in a modest reduction in both G' and G'', suggesting a slight softening of the gel network. However, this did not alter the fundamental viscoelastic profile where elasticity dominates, ensuring the adhesive performance and mechanical stability are preserved. Notably, both formulations exhibited pronounced shear-thinning behaviour, as evidenced by the significant reduction in complex viscosity with increasing frequency. This property ensures while the gel can momentarily accommodate shear stress, such as during application or repositioning, it rapidly regains viscosity once in place, thereby maintaining stable adhesion to the wound area. Similar trends have been reported previously: binary protein-polysaccharide hydrogels demonstrated a frequency-dependent decrease in complex viscosity, confirming their shear-thinning characteristics, which is typical of viscoelastic materials [31]. Further, a study on carbomer 940-based hydrogels also showed a significant reduction in viscosity with increasing frequency, consistent with behaviour of viscoelastic semi-solids [32]. The slightly higher initial viscosity observed with naringenin-loaded gels may confer additional handling benefits, offering a firmer yet still compliant dressing. Overall, these rheological attributes support potential of the naringenin-loaded hydrogel as a mechanically stable and adhesive dressing, capable of conforming to the wound area while resisting dislodgement during wear.

Adhesive performance of hydrogels is a critical parameter for consideration in clinical application as wound dressings, where secure yet gentle adhesion is needed to maintain contact with the wound area without causing trauma upon removal. The ball-tack test provided a practical measure of this by quantifying both maximum pull-off force during detachment and minimum force during initial contact. Findings demonstrated incorporation of 0.02% w/w naringenin did not significantly affect hydrogels adhesive strength compared to the unloaded formulation. This suggests therapeutic loading up to this concentration at least does not compromise the adhesive integrity required for clinical use. Similarly, the minimum force recorded during loading, indicative of hydrogel ability to conform to the surface, showed no significant difference between formulations. This implies naringenin would not hinder hydrogel ability to establish contact with skin, an essential feature for effective adhesion and drug delivery. Together, these results confirm drug loading did not detract from the mechanical adhesive properties, supporting suitability of these formulations for wound dressing applications.

The swelling behaviour of the Na-AMPS-based hydrogels, reaching equilibrium within 30 min, provides a hydrated network capable of absorbing and retaining water, which is critical for wound dressings. Similar rapid swelling behaviour, with complete hydration achieved in under 1 h, has been reported in previous studies of Na-AMPS hydrogels [22]. This hydrated and stable gel matrix likely contributes to the controlled release characteristics observed [22]. The release profile observed in this study confirms the ability of the Na-AMPS-based gel formulation to provide sustained and controlled delivery of naringenin. In contrast to the immediate and complete release observed from naringenin in solution, the gel significantly slowed the release rate over 24 h without any evidence of burst release. Comparable findings have been reported in studies incorporating naringenin into more complex systems such as liposomal gels, where the addition of lipid carriers and polymer matrices reduced the rate of drug release [13]. Notably, the present formulation achieves a similarly controlled release using a single-polymer system, underscoring the potential of Na-AMPS hydrogels as a simple and effective platform for sustained topical or transdermal drug delivery. Such a release profile is desirable for maintaining prolonged local drug availability and minimising dosing frequency, which may ultimately enhance therapeutic outcomes and patient compliance. We observed gel swelling throughout the release period, consistent with previous reports that also highlight the potential of such systems for wound-related applications, including as burn dressings [18]. In related studies, drug release from Na-AMPS-based hydrogels has been shown an initial fast release followed by a slower, diffusion-controlled phase [22]. This has been attributed to both a reduction in the drug concentration gradient over time and specific interactions between the drug and the polymer network. For instance, the incomplete release of ciprofloxacin and silver sulfadiazine from Na-AMPS/PCLDA hydrogels was attributed to hydrogen bonding and electrostatic interactions between functional groups on the drug molecules and the polymer matrix [22]. Similar mechanisms may also contribute to the sustained release of naringenin observed in this study, suggesting that both diffusional control and polymer–drug interactions play a role in modulating its release kinetics. Such features further support the utility of Na-AMPS hydrogels in delivering poorly soluble, bioactive compounds in a controlled and therapeutically favourable manner. Further studies in vivo are required to understand drug release into wound exudate followed by further formulation optimisation.

The cytotoxicity assessment of permeate from in vitro naringenin release studies in HUVEC and HDFa cells provides important insights into formulation safety profile for therapeutic use. Both cell types maintained high viability (> 90%) at submicromolar concentrations (0.1–0.5 μM), indicating naringenin is well tolerated within this range. However, significant, dose-dependent reductions in viability occurred at ≥ 1 μM, confirming a narrow therapeutic window. This is consistent with previous studies reporting reduced HDFa viability with increasing naringenin concentrations greater than 1 μM [13]. Together, these findings highlight the importance of dose control in formulation strategies, especially for topical or localised delivery via adhesive hydrogels, where submicromolar concentrations should be maintained to avoid cytotoxicity while retaining therapeutic potential.

Naringenin demonstrated significant antioxidant and anti-inflammatory effects in endothelial and dermal fibroblast cells under diabetic-like stress conditions, supporting its therapeutic potential in wound healing. Both HUVEC and HDFa cells exposed to hyperglycaemic and pro-inflammatory environments exhibited a marked increase in ROS production, consistent with elevated oxidative stress commonly associated with diabetes. Naringenin treatment significantly reduced ROS levels in both cell types, though not to baseline, reflecting a partial but meaningful antioxidant effect. This aligns with previous studies highlighting naringenin’s capacity to scavenge free radicals and modulate oxidative pathways, which are critical in mitigating diabetic complications [33, 34]. Further, TMRM staining revealed mitochondrial membrane potential—typically disrupted under inflammatory stress—was partially restored following naringenin treatment, indicating a protective effect on mitochondrial function. These findings are in line with previous reports in neuronal cells, where naringenin increased high-energy phosphate levels, enhanced adenine nucleotide translocase transport activity, and significantly elevated mitochondrial membrane potential under oxidative stress [35]. These effects have been attributed to activation of the Nrf2/ARE signalling pathway, which plays a role in cellular antioxidant defences and mitochondrial homeostasis [35]. Thus, naringenin may support wound healing not only through ROS suppression but also via preservation of mitochondrial function. Further optimisation of dose and release kinetics could enhance such protective effects.

Naringenin also effectively attenuated IL-6 secretion in both cell types under inflammatory stress, demonstrating its anti-inflammatory activity. This reduction in IL-6 is particularly relevant, as chronic inflammation driven by elevated pro-inflammatory cytokines impairs wound healing in diabetes [36]. Similar anti-inflammatory effects of naringenin have been reported in vivo. A study investigating the molecular basis of naringenin’s action in a dextran sulphate sodium-induced murine colitis model and found that it significantly reduced IL-6 mRNA expression by inhibiting NF-κB p65 activation in macrophages [37]. In a separate study using LPS- and Con A–induced murine models of liver injury, naringenin protected against cytokine-induced organ damage and lethality [37]. Interestingly, this protective effect occurred without affecting TLR signalling or mRNA stability, and instead was attributed to enhanced intracellular cytokine degradation via lysosome- and TFEB-dependent pathways, suggesting a post-translational regulatory mechanism. In addition, the wound healing scratch assay demonstrated naringenin significantly improved endothelial cell migration under TNF-α–induced stress, indicating that suppression of pro-inflammatory signalling may directly contribute to enhanced wound closure. This is particularly relevant in the context of diabetic wounds, where chronic inflammation is a major barrier to repair and contributes to poor healing outcomes despite advanced therapeutic options [38]. Collectively, these findings indicate naringenin can modulate inflammatory responses suggesting its therapeutic potential in restoring a diabetic wound microenvironment conducive to tissue repair.

In HDFa cells, naringenin also modulated matrix remodelling by significantly lowering MMP-9 levels, which were otherwise elevated under diabetic-like conditions. Excessive MMP-9 is associated with matrix degradation and delayed tissue regeneration, thus, its suppression suggests naringenin may help restore matrix homeostasis and modulate inflammatory activity, consistent with previous reports linking MMP-9 suppression to anti-inflammatory effects [39–41]. Additionally, the reduction in TGF-β secretion, while still elevated compared to controls, indicates that naringenin can temper the excessive fibrotic response typical of diabetic wounds [42]. Collectively, these findings suggest naringenin exerts multi-modal protective effects by reducing oxidative stress, inflammation, and aberrant matrix remodelling whilst improving mitochondrial function, supporting its potential incorporation into hydrogel-based therapies for diabetic wound management.

This study provides initial evidence supporting the potential of a naringenin-loaded Na-AMPS hydrogel for diabetic wound management; however, the findings are limited to in vitro models, and in vivo studies are needed to confirm therapeutic efficacy, drug release behaviour, and biocompatibility in the more complex wound environment. Mechanical robustness and long-term stability may also benefit from further optimisation. As bacterial infection is a major contributor to delayed healing in diabetic foot ulcers, future studies should investigate the effects of this formulation on clinically relevant wound pathogens. Understanding its antimicrobial performance will be essential to guide formulation refinement and ensure the hydrogel can address both the biological and microbiological challenges of diabetic wound care.

## Conclusion

Chronic diabetic wounds are influenced by multiple pathological factors, including oxidative stress, persistent inflammation, and impaired matrix remodelling, which collectively contribute to delayed tissue repair. Conventional dressings often fail to address these underlying challenges, highlighting the need for treatments that can both protect the wound and deliver therapeutic agents locally. This study demonstrates the potential of a Na-AMPS-based hydrogel as a platform for sustained delivery of naringenin, an antioxidant compound, in wound healing applications. The formulation exhibited favourable viscoelastic and adhesive properties, incorporation of naringenin did not compromise structural integrity and conformability required for topical dressings. Further, the hydrogel enabled controlled release of naringenin with up to one fifth of total naringenin released over 24 h, in contrast to the rapid release observed from solution. Cytotoxicity testing indicated that naringenin is well tolerated at submicromolar concentrations, which also coincided with significant biological activity. Under diabetic-like stress conditions in both HUVEC and HDFa cells, naringenin reduced oxidative stress and attenuated IL-6 as well as partially restored mitochondrial function. Further, in HDFa cells, naringenin reduced MMP-9 secretion and moderated excessive TGF-β production, collectively supporting an environment more conducive to tissue repair. Finally, naringenin was able to support cell migration in a HUVEC wound healing assay. These findings highlight the promise of naringenin-loaded Na-AMPS hydrogels as adhesive dressings for improved diabetic wound management.

## Supplementary Information

Below is the link to the electronic supplementary material.

ESM 1 (DOCX 95.7 KB)

## Data Availability

The datasets generated and/or analysed during the current study are not publicly available due to ongoing related studies but are available from the corresponding author on reasonable request.
