# Supplementary material for: Naringenin Loaded Hydrogel Supports Wound Repair in a Cell Model of Diabetic Skin
Source: Pharm Res. 2026 Jan 31;43(3):833–47. doi: 10.1007/s11095-026-04029-z (PMC13076458; doi:10.1007/s11095-026-04029-z)
Supplement: Supplementary file 1 — (DOCX 95.7 KB) [file 11095_2026_4029_MOESM1_ESM.docx]

# Supplementary Figure

Supplementary Figure 1. A representative force-displacement curve for an adhesive hydrogel loaded with naringenin. Each curve illustrates the measurement from one sample and one time.
